# Severe Thrombocytopenia With Wet Purpura in Brucellosis: A Case Report

**DOI:** 10.1002/ccr3.72838

**Published:** 2026-06-02

**Authors:** Fatemeh Ravanbakhsh Ghavghani, Hamid Oveisi Oskouei, Hamed Ebrahimzadeh Leylabadlo

**Affiliations:** ^1^ Department of Infectious Diseases, Faculty of Medicine Tabriz University of Medical Sciences Tabriz Iran; ^2^ Infectious and Tropical Diseases Research Center Tabriz University of Medical Sciences Tabriz Iran; ^3^ Liver and Gastrointestinal Diseases Research Center Tabriz University of Medical Sciences Tabriz Iran

**Keywords:** *brucella* spp., brucellosis, immune thrombocytopenic purpura, thrombocytopenia

## Abstract

Brucellosis is a zoonotic infection that passes to humans from infected animals via direct contact or the intake of raw milk and its products. The hematologic profile most frequently seen in acute brucellosis includes mild anemia and leukopenia. Severe form of thrombocytopenia and multiple wet purpura over the tongue and buccal mucosa are less frequently reported. This article describes a case of a 24‐year‐old man who presented with an absolute platelet count of 1000 × 10^3^/L; multiple wet purpura over the tongue and buccal mucosa and petechial lesions on the distal shins. The patient was diagnosed with brucellosis according to a positive serologic test and was treated with an antibiotic regimen including ceftriaxone and doxycycline, dexamethasone and a transfusion of 6 units of platelets. Mucocutaneous lesions resolved after 3 days of hospitalization, and platelet counts showed a steady upward trend. Brucella infection may cause severe thrombocytopenia and wet purpura over the tongue and buccal mucosa, which can be reversed with appropriate antimicrobials and steroids.

## Key Clinical Message

Brucellosis can present with severe, isolated thrombocytopenia and mucosal hemorrhagic bullae mimicking hematologic malignancy. Early recognition and treatment with antibiotics and corticosteroids leads to rapid platelet recovery and resolution of purpura, obviating the need for invasive diagnostic procedures.

## 1 Introduction

Brucellosis is a common zoonotic disease worldwide, affecting both animals and humans and causing significant economic and public health impacts [1]. Brucellosis is transmitted to humans directly or indirectly from infected animals [2]. Brucellosis is still a major public health issue across much of the Middle East, responsible for many new infections annually [3]. The disease is endemic in most parts of Iran, and the annual incidence of both human and animal brucellosis remains high [4]. The clinical manifestations of brucellosis exhibit variability and frequently encompass undulating fevers, chills, myalgia, nausea, vomiting, diarrhea, and headaches [5]. The disease causes a variety of hematological abnormalities, including anemia, leukopenia, thrombocytopenia, and pancytopenia; however, thrombocytopenia and pancytopenia have been less frequent during the clinical course [6]. Thrombocytopenia is often seen in these patients, but severe cases resulting in mucosal bleeding or wet purpura are rare [7]. The presence of wet purpura in patients with thrombocytopenia is a sinister sign that indicates imminent bleeding and requires aggressive therapeutic intervention [8]. The mechanism of brucellosis that leads to thrombocytopenia is not fully understood and may involve multiple factors [9]. These factors could include the immune destruction of platelets, disseminated intravascular coagulopathy, secondary hemophagocytic lymphohistiocytosis, hypersplenism, and bone marrow suppression [10]. Hereby, we describe a patient with severe thrombocytopenia and wet purpura on the tongue and buccal mucosa, which was diagnosed as Brucellosis.

## 2 Case History

**FIGURE 1:**
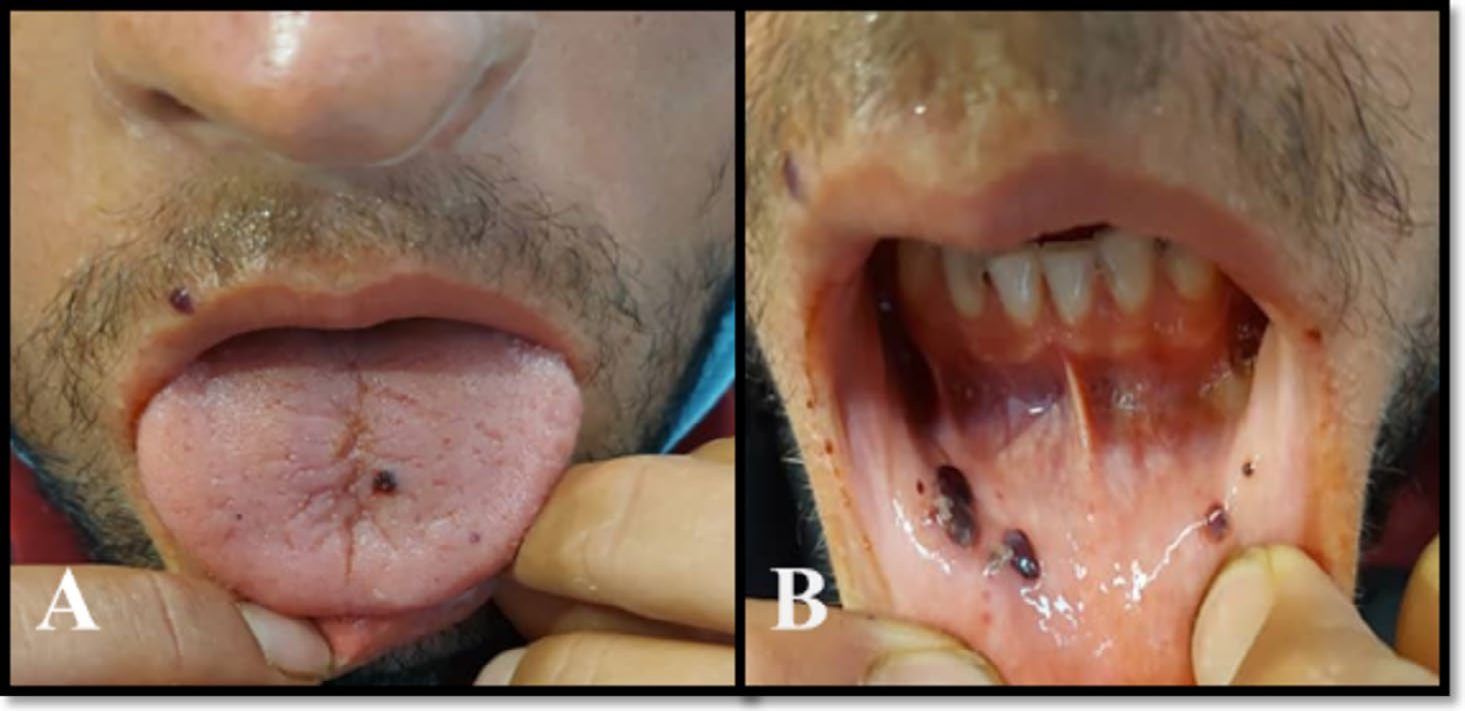
(A) Clinical photograph showing wet purpura over the tongue on Day 1 of hospital visit. (B) Clinical photograph showing wet purpura over the buccal mucosa on Day 1 of hospital visit.

**FIGURE 2:**
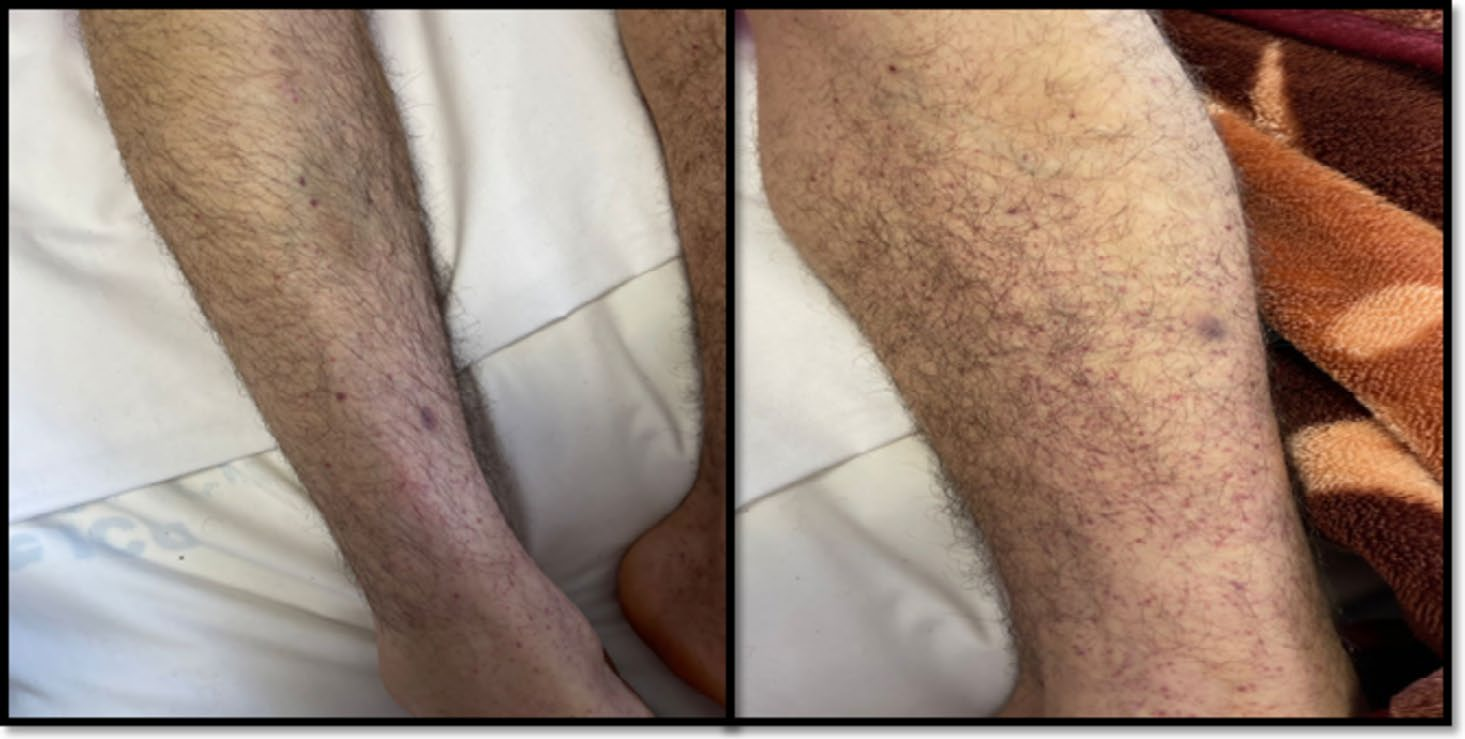
Clinical photograph showing petechial lesions on the distal shins of both legs.

A previously healthy 24‐year‐old man presented to the infectious disease clinic at Sina Hospital, Iran, with a three‐day history of oral lesions. He had been taking cotrimoxazole and metronidazole for 3 days without improvement. The patient did not mention fever, chills, cough, or headache over the past 3 days or at the time of the visit. There was wet purpura over the tongue and buccal mucosa (Figure 1). He had mild sweating on his forehead and petechial lesions on the distal shins of both legs (Figure 2).

## 3 Differential Diagnosis, Investigations, and Treatment

With the patient having arrived from an endemic region and given his and his father's occupation as livestock farmers, a complete blood count (CBC) and a Brucella serology test were requested. Initial CBC suggested severe thrombocytopenia with platelets 1000 × 10^3^/L. The Wright test was performed on the patient, and an observed titer of 1:640 was obtained. The Combs Wright test also showed a titer of 1:1320. The 2‐Mercaptoethanol (2ME) titer was 1:320. Therefore, the patient was hospitalized to receive an antibiotic regimen and close monitoring for initiation. The patient's vital signs were within normal limits upon admission and included: blood pressure 120/75 mmHg, heart rate 80 bpm, respiratory rate 18 bpm, temperature 36.6°C, oxygen saturation 97%. An abdominal ultrasonography showed evidence of hepatosplenomegaly, revealing an enlarged spleen measuring 163 mm.

Hepatocellular pattern showed elevated aminotransferases (AST 194 U/L, ALT 139 U/L) and a markedly high lactate dehydrogenase (LDH 1457 U/L). Other laboratory parameters were as follows: PT 13 s, PTT 30 s, INR 1, Urea 32 mg/dL, Creatinine 1.0 mg/dL, CRP 2 mg/L, ESR 15 mm/h, all within normal limits.

Initially, an anti‐brucellosis treatment regimen consisting of intravenous ceftriaxone, oral doxycycline (100 mg every 12 h), and dexamethasone (4 mg every 12 h for 3 days) along with a transfusion of 6 units of platelets was administered. The significant clinical improvement was observed until the third day of hospitalization; mucocutaneous lesions were resolved, and platelet counts showed a steady upward trend. The increase in the patient's platelet count between Days 3 and 12 was as follows: 4000, 9000, 15,000, 21,000, 41,000, 42,000, 45,000, 49,000, 50,000, and 61,000/μL.

The liver enzymes had substantially decreased by Day 12 to AST 35 U/L and ALT 81 U/L, and also by Day 4 lactate dehydrogenase decreased to 881 U/L. The autoimmune serological tests, such as ANA, anti‐dsDNA, ANCA, and complement (C3, C4, CH50) were negative. Alternative causes of thrombocytopenia were ruled out according to the clinical context and the patient's impressive response to anti‐Brucella therapy. The patient's antibiotic regimen transitioned to oral doxycycline and rifampin on Day 10 and was discharged in stable condition on Day 13. The patient was advised to continue the treatment for 2 months and then discontinue it. The patient's follow up 6 months and 1 year later confirmed complete and sustained recovery, with all hematological and biochemical parameters remaining normal.

## 4 Conclusion and Follow‐Up

Here, we present a case of severe thrombocytopenia with wet purpura over the tongue and the buccal mucosa on admission to our hospital, which underwent brucellosis treatment planning. In conclusion, our case is a rare wet purpura with severe thrombocytopenia due to brucellosis. Hence, this case may suggest that brucellosis should be considered among the causes of wet purpura in patients with severe thrombocytopenia. Also, given that brucellosis is common in some parts of Iran, severe thrombocytopenia and purpura should also be considered as potential complications of this disease, especially in endemic areas and among individuals engaged in livestock farming.

## 5 Discussion

Brucellosis remains a neglected health issue in low‐ and middle‐income nations. The disease can become severe, debilitating, or chronic, though death is a rare outcome [11, 12]. The distribution of brucellosis varies, depending on time and region [13]. In Iran, brucellosis cases are recognized as endemic in many parts and report a significant annual incidence rate [14]. Hematological abnormalities in brucellosis have been reported in the literature. There are several hematological manifestations in brucellosis, including thrombocytopenia, TTP, anemia, hemophagocytic syndrome, leukopenia, and DIC [15]. Among the various clinical manifestations, thrombocytopenia may be found in 3%–26% of brucellosis cases [16]. In some cases, thrombocytopenia can be serious and can be associated with bleeding into the skin (purpura) and from mucosal sites [17]. In some patients, thrombocytopenic purpura may appear together with constitutional symptoms which can also include fever, malaise, arthritis, and hepatosplenomegaly [18]. Studies suggest that the absence of bleeding in severe thrombocytopenia can be attributed to preserved platelet function, not just the platelet count itself [19]. Intravenous immunoglobulin (IVIG) has been reported as an effective urgent therapy in cases of brucellosis‐induced severe thrombocytopenic purpura. The pathogenesis of thrombocytopenia in brucellosis remains unclear, but some different mechanisms have been described, including bone marrow suppression, hypersplenism, and immune mechanism [20].

Pancytopenia usually presents with symptoms of bone marrow failure and hypersplenism [21]. In the present study case, there was evidence of bone marrow suppression, according to the WBC, Hb, platelets, and reticulocyte count. Also, there was evidence of hepatosplenomegaly and an enlarged spleen. To exclude other causes of thrombocytopenia, the serological assays for autoimmune markers—including ANA, anti‐dsDNA, ANCA, and complement—were undertaken, and all results were within normal limits.

Brucellosis treatment is directed at disease control and prevention of complications, including relapses, sequelae, and death [22]. Infectious thrombocytopenia should generally be treated by treating the underlying infection. This was also true in our case; with the initiation of antibiotic treatment, mucocutaneous lesions resolved, and platelet counts showed a steady upward trend. In a study by Young, two patients with brucellosis and thrombocyte counts of 3000/mL and 5000/mL were treated with platelet transfusion, fresh‐frozen plasma, steroids, and antibacterial drugs. However, one of the patients died due to intracranial bleeding [23]. Also, other treatment options have also been reported for severe thrombocytopenia associated with brucellosis. Intravenous immunoglobulin (IVIG) has been reported as an effective urgent therapy in cases of brucellosis‐induced severe thrombocytopenic purpura [24].

## Author Contributions

**Fatemeh Ravanbakhsh Ghavghani:** investigation, project administration, writing – original draft, writing – review and editing. **Hamid Oveisi Oskouei:** conceptualization, data curation, investigation, writing – original draft, writing – review and editing. **Hamed Ebrahimzadeh Leylabadlo:** supervision, writing – original draft, writing – review and editing.

## Funding

The authors have nothing to report.

## Ethics Statement

The ethical committee approval number IR.TBZMED.REC.1403.093.

## Consent

Written informed consent was obtained from the patient for participation in this intervention and publication of associated clinical data with the understanding that this information is publicly available.

## Conflicts of Interest

The authors declare no conflicts of interest.

## Data Availability

The data that support the findings of this study are available from the corresponding author upon reasonable request.
